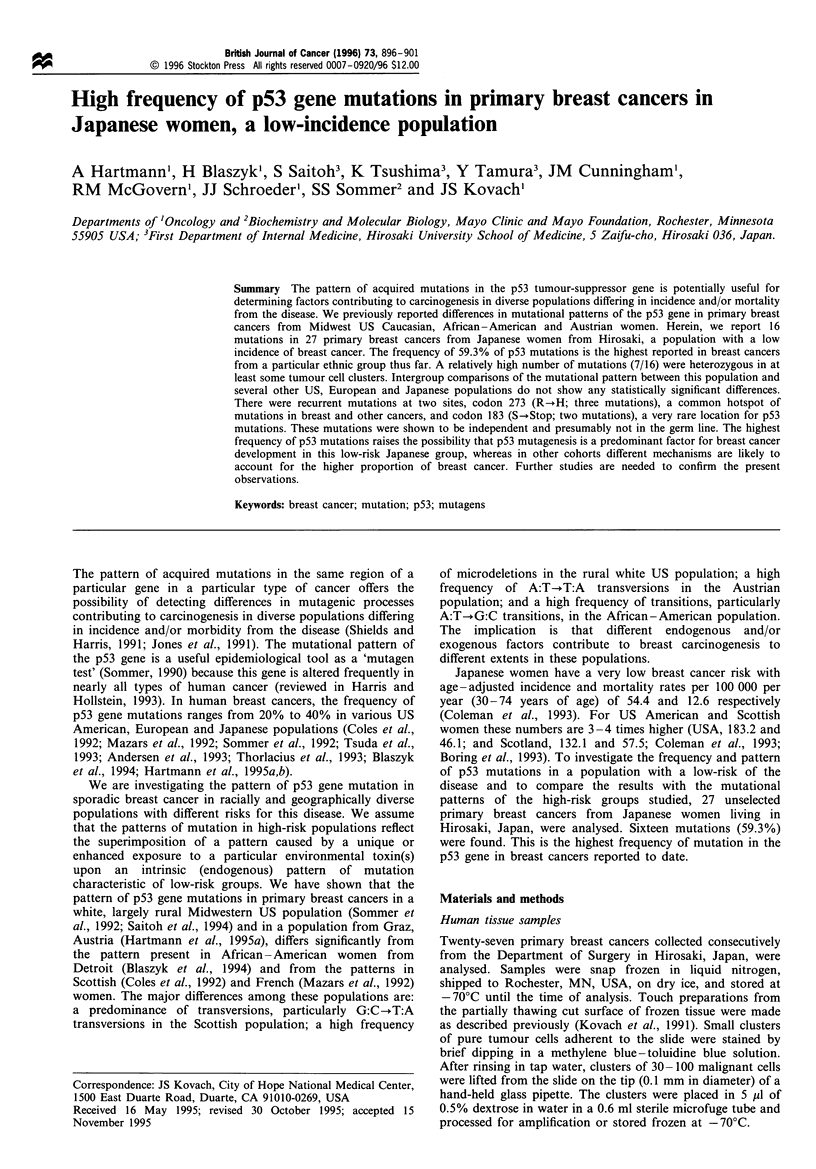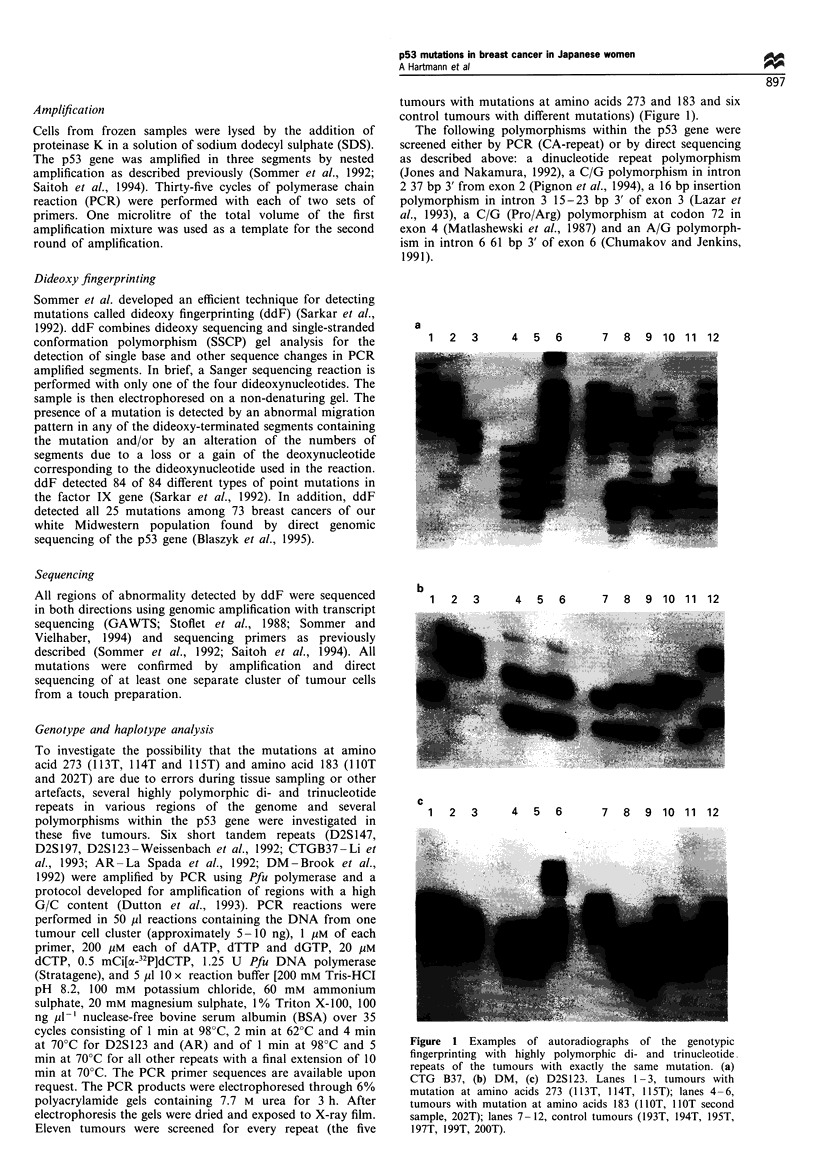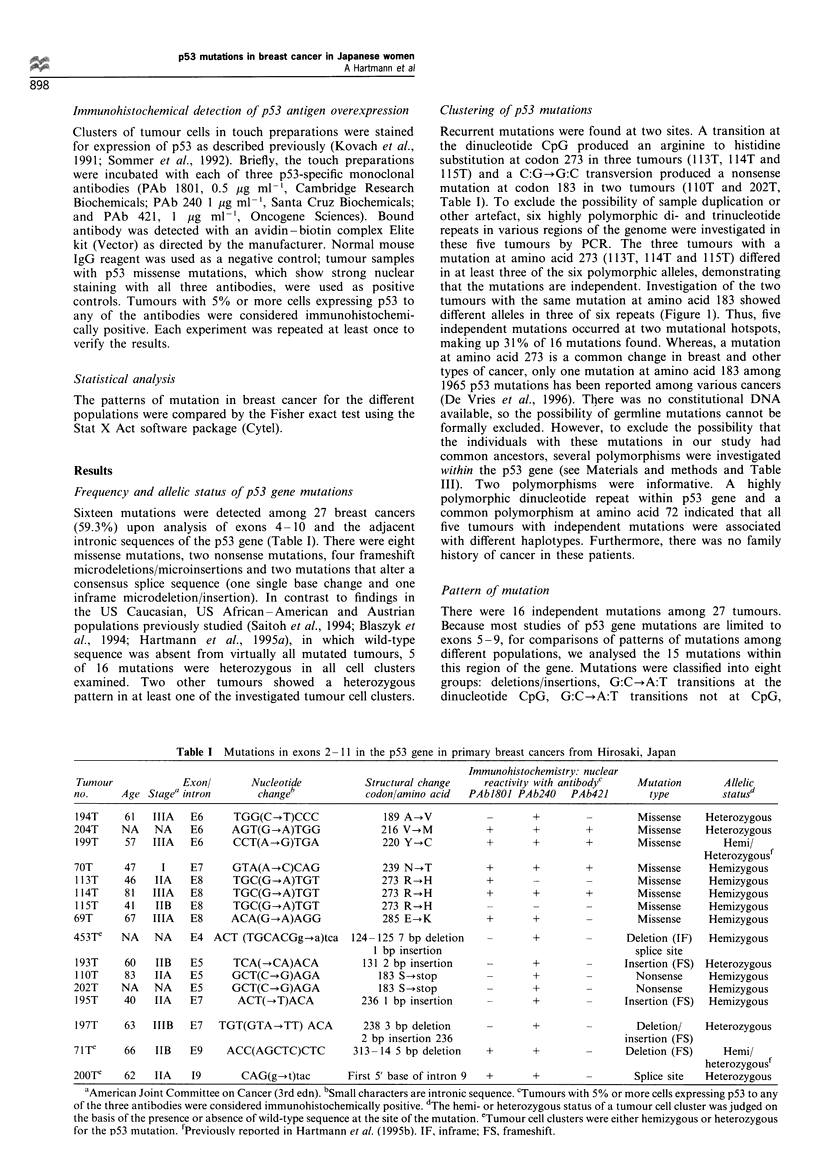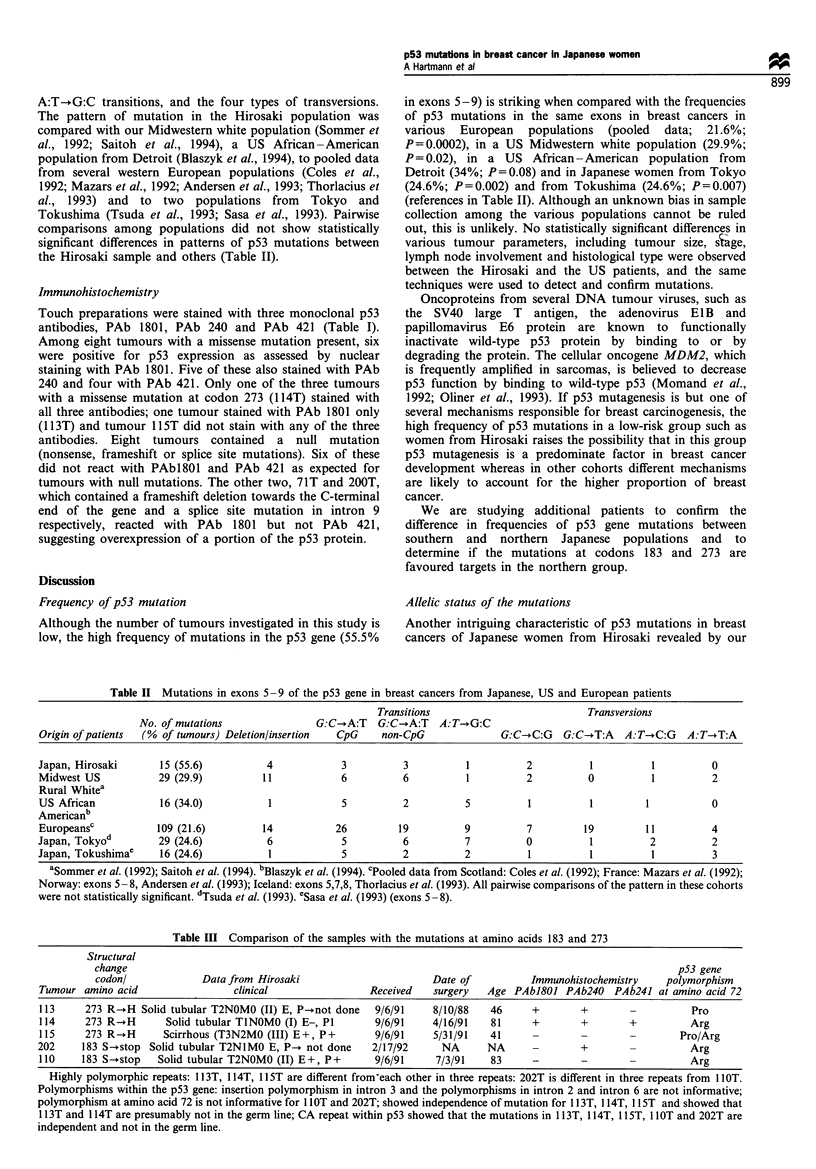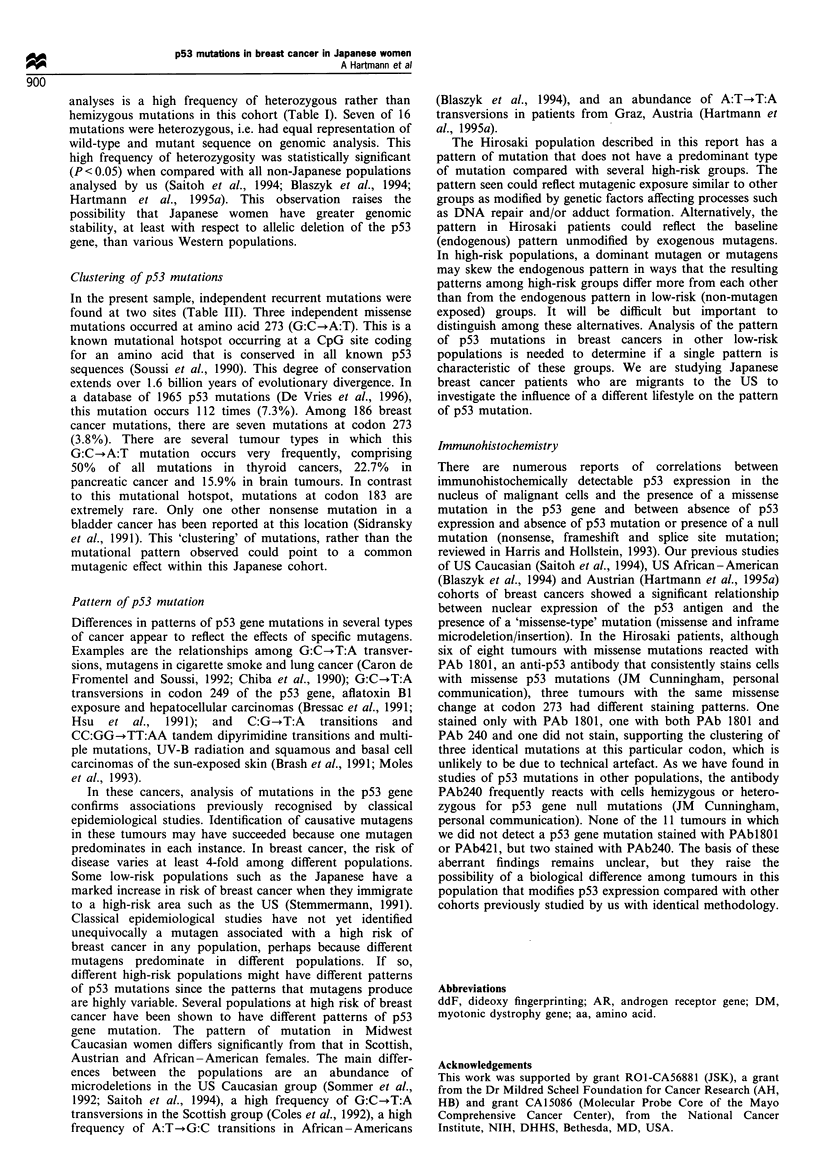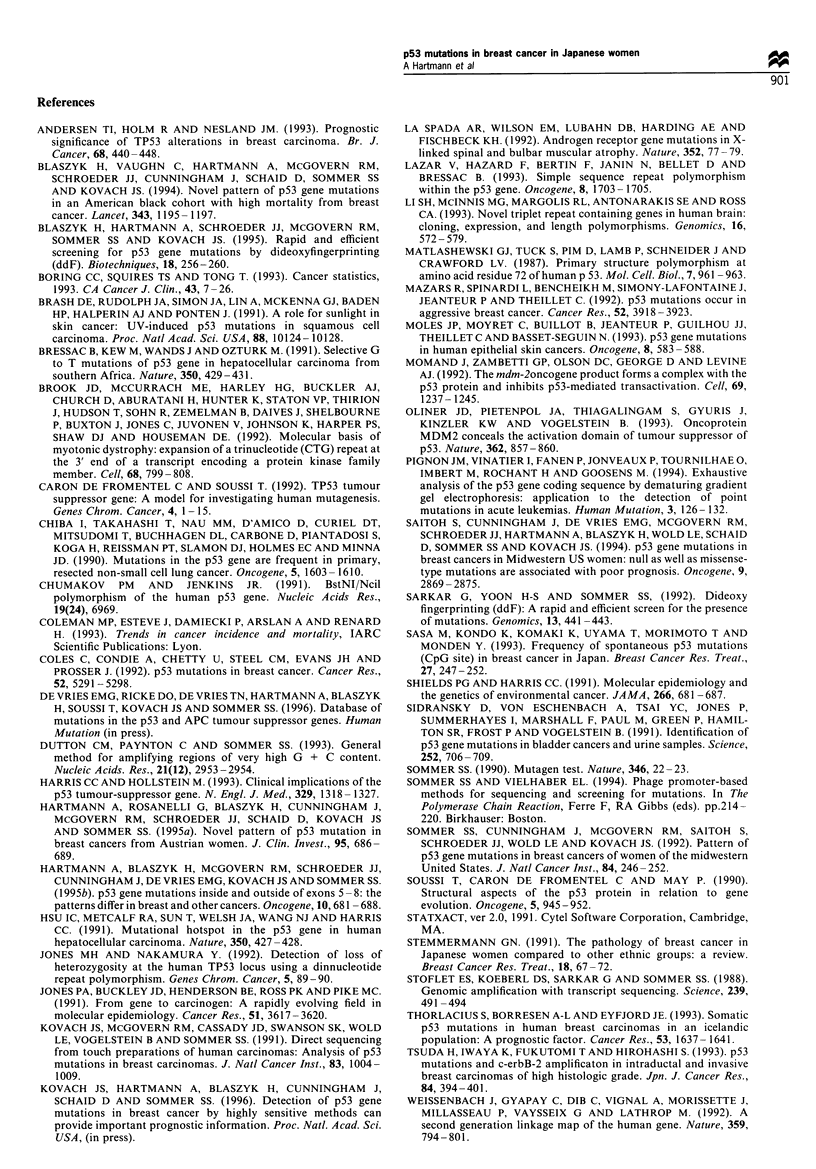# High frequency of p53 gene mutations in primary breast cancers in Japanese women, a low-incidence population.

**DOI:** 10.1038/bjc.1996.179

**Published:** 1996-04

**Authors:** A. Hartmann, H. Blaszyk, S. Saitoh, K. Tsushima, Y. Tamura, J. M. Cunningham, R. M. McGovern, J. J. Schroeder, S. S. Sommer, J. S. Kovach

**Affiliations:** Department of Oncology, Mayo Clinic, Rochester, Minnesota 55905, USA.

## Abstract

**Images:**


British Journal of Cancer (1996) 73, 896-901
© 1996 Stockton Press All rights reserved 0007-0920/96 $12.00

### High frequency of p53 gene mutations in primary breast cancers in Japanese women, a low-incidence population

A Hartmann[1], H Blaszyk[1], S Saitoh[3], K Tsushima[3], Y Tamura[3], JM Cunningham[1], RM McGovern[1], JJ Schroeder[1], SS Sommer[2] and JS Kovach[1]

*Departments of [1]Oncology and [2]Biochemistry and Molecular Biology, Mayo Clinic and Mayo Foundation, Rochester, Minnesota 55905 USA; [3]First Department of Internal Medicine, Hirosaki University School of Medicine, 5 Zaifu-cho, Hirosaki 036, Japan.*

**Summary** The pattern of acquired mutations in the p53 tumour-suppressor gene is potentially useful for determining factors contributing to carcinogenesis in diverse populations differing in incidence and/or mortality from the disease. We previously reported differences in mutational patterns of the p53 gene in primary breast cancers from Midwest US Caucasian, African-American and Austrian women. Herein, we report 16 mutations in 27 primary breast cancers from Japanese women from Hirosaki, a population with a low incidence of breast cancer. The frequency of 59.3% of p53 mutations is the highest reported in breast cancers from a particular ethnic group thus far. A relatively high number of mutations (7/16) were heterozygous in at least some tumour cell clusters. Intergroup comparisons of the mutational pattern between this population and several other US, European and Japanese populations do not show any statistically significant differences. There were recurrent mutations at two sites, codon 273 (R→H; three mutations), a common hotspot of mutations in breast and other cancers, and codon 183 (S→Stop; two mutations), a very rare location for p53 mutations. These mutations were shown to be independent and presumably not in the germ line. The highest frequency of p53 mutations raises the possibility that p53 mutagenesis is a predominant factor for breast cancer development in this low-risk Japanese group, whereas in other cohorts different mechanisms are likely to account for the higher proportion of breast cancer. Further studies are needed to confirm the present observations.

**Keywords:** breast cancer; mutation; p53; mutagens

The pattern of acquired mutations in the same region of a particular gene in a particular type of cancer offers the possibility of detecting differences in mutagenic processes contributing to carcinogenesis in diverse populations differing in incidence and/or morbidity from the disease (Shields and Harris, 1991; Jones *et al.*, 1991). The mutational pattern of the p53 gene is a useful epidemiological tool as a 'mutagen test' (Sommer, 1990) because this gene is altered frequently in nearly all types of human cancer (reviewed in Harris and Hollstein, 1993). In human breast cancers, the frequency of p53 gene mutations ranges from 20% to 40% in various US American, European and Japanese populations (Coles *et al.*, 1992; Mazars *et al.*, 1992; Sommer *et al.*, 1992; Tsuda *et al.*, 1993; Andersen *et al.*, 1993; Thorlacius *et al.*, 1993; Blaszyk *et al.*, 1994; Hartmann *et al.*, 1995*a,b*).

We are investigating the pattern of p53 gene mutation in sporadic breast cancer in racially and geographically diverse populations with different risks for this disease. We assume that the patterns of mutation in high-risk populations reflect the superimposition of a pattern caused by a unique or enhanced exposure to a particular environmental toxin(s) upon an intrinsic (endogenous) pattern of mutation characteristic of low-risk groups. We have shown that the pattern of p53 gene mutations in primary breast cancers in a white, largely rural Midwestern US population (Sommer *et al.*, 1992; Saitoh *et al.*, 1994) and in a population from Graz, Austria (Hartmann *et al.*, 1995*a*), differs significantly from the pattern present in African-American women from Detroit (Blaszyk *et al.*, 1994) and from the patterns in Scottish (Coles *et al.*, 1992) and French (Mazars *et al.*, 1992) women. The major differences among these populations are: a predominance of transversions, particularly G:C→T:A transversions in the Scottish population; a high frequency of microdeletions in the rural white US population; a high frequency of A:T→T:A transversions in the Austrian population; and a high frequency of transitions, particularly A:T→G:C transitions, in the African-American population. The implication is that different endogenous and/or exogenous factors contribute to breast carcinogenesis to different extents in these populations.

Japanese women have a very low breast cancer risk with age-adjusted incidence and mortality rates per 100 000 per year (30-74 years of age) of 54.4 and 12.6 respectively (Coleman *et al.*, 1993). For US American and Scottish women these numbers are 3-4 times higher (USA, 183.2 and 46.1; and Scotland, 132.1 and 57.5; Coleman *et al.*, 1993; Boring *et al.*, 1993). To investigate the frequency and pattern of p53 mutations in a population with a low-risk of the disease and to compare the results with the mutational patterns of the high-risk groups studied, 27 unselected primary breast cancers from Japanese women living in Hirosaki, Japan, were analysed. Sixteen mutations (59.3%) were found. This is the highest frequency of mutation in the p53 gene in breast cancers reported to date.

### Materials and methods

### *Human tissue samples*

Twenty-seven primary breast cancers collected consecutively from the Department of Surgery in Hirosaki, Japan, were analysed. Samples were snap frozen in liquid nitrogen, shipped to Rochester, MN, USA, on dry ice, and stored at −70°C until the time of analysis. Touch preparations from the partially thawing cut surface of frozen tissue were made as described previously (Kovach *et al.*, 1991). Small clusters of pure tumour cells adherent to the slide were stained by brief dipping in a methylene blue-toluidine blue solution. After rinsing in tap water, clusters of 30-100 malignant cells were lifted from the slide on the tip (0.1 mm in diameter) of a hand-held glass pipette. The clusters were placed in 5 μl of 0.5% dextrose in water in a 0.6 ml sterile microfuge tube and processed for amplification or stored frozen at −70°C.

Correspondence: JS Kovach, City of Hope National Medical Center, 1500 East Duarte Road, Duarte, CA 91010-0269, USA
Received 16 May 1995; revised 30 October 1995; accepted 15 November 1995

*Amplification*

Cells from frozen samples were lysed by the addition of proteinase K in a solution of sodium dodecyl sulphate (SDS). The p53 gene was amplified in three segments by nested amplification as described previously (Sommer *et al.*, 1992; Saitoh *et al.*, 1994). Thirty-five cycles of polymerase chain reaction (PCR) were performed with each of two sets of primers. One microlitre of the total volume of the first amplification mixture was used as a template for the second round of amplification.

*Dideoxy fingerprinting*

Sommer *et al.* developed an efficient technique for detecting mutations called dideoxy fingerprinting (ddF) (Sarkar *et al.*, 1992). ddF combines dideoxy sequencing and single-stranded conformation polymorphism (SSCP) gel analysis for the detection of single base and other sequence changes in PCR amplified segments. In brief, a Sanger sequencing reaction is performed with only one of the four dideoxynucleotides. The sample is then electrophoresed on a non-denaturing gel. The presence of a mutation is detected by an abnormal migration pattern in any of the dideoxy-terminated segments containing the mutation and/or by an alteration of the numbers of segments due to a loss or a gain of the deoxynucleotide corresponding to the dideoxynucleotide used in the reaction. ddF detected 84 of 84 different types of point mutations in the factor IX gene (Sarkar *et al.*, 1992). In addition, ddF detected all 25 mutations among 73 breast cancers of our white Midwestern population found by direct genomic sequencing of the p53 gene (Blaszyk *et al.*, 1995).

*Sequencing*

All regions of abnormality detected by ddF were sequenced in both directions using genomic amplification with transcript sequencing (GAWTS; Stoflet *et al.*, 1988; Sommer and Vielhaber, 1994) and sequencing primers as previously described (Sommer *et al.*, 1992; Saitoh *et al.*, 1994). All mutations were confirmed by amplification and direct sequencing of at least one separate cluster of tumour cells from a touch preparation.

*Genotype and haplotype analysis*

To investigate the possibility that the mutations at amino acid 273 (113T, 114T and 115T) and amino acid 183 (110T and 202T) are due to errors during tissue sampling or other artefacts, several highly polymorphic di- and trinucleotide repeats in various regions of the genome and several polymorphisms within the p53 gene were investigated in these five tumours. Six short tandem repeats (D2S147, D2S197, D2S123-Weissenbach *et al.*, 1992; CTGB37-Li *et al.*, 1993; AR-La Spada *et al.*, 1992; DM-Brook *et al.*, 1992) were amplified by PCR using *Pfu* polymerase and a protocol developed for amplification of regions with a high G/C content (Dutton *et al.*, 1993). PCR reactions were performed in 50 $\mu$l reactions containing the DNA from one tumour cell cluster (approximately 5-10 ng), 1 $\mu$M of each primer, 200 $\mu$M each of dATP, dTTP and dGTP, 20 $\mu$M dCTP, 0.5 mCi[$\alpha$-$^{32}$P]dCTP, 1.25 U *Pfu* DNA polymerase (Stratagene), and 5 $\mu$l 10 × reaction buffer [200 mM Tris-HCI pH 8.2, 100 mM potassium chloride, 60 mM ammonium sulphate, 20 mM magnesium sulphate, 1% Triton X-100, 100 ng $\mu l^{-1}$ nuclease-free bovine serum albumin (BSA) over 35 cycles consisting of 1 min at 98°C, 2 min at 62°C and 4 min at 70°C for D2S123 and (AR) and of 1 min at 98°C and 5 min at 70°C for all other repeats with a final extension of 10 min at 70°C. The PCR primer sequences are available upon request. The PCR products were electrophoresed through 6% polyacrylamide gels containing 7.7 M urea for 3 h. After electrophoresis the gels were dried and exposed to X-ray film. Eleven tumours were screened for every repeat (the five tumours with mutations at amino acids 273 and 183 and six control tumours with different mutations) (Figure 1).

The following polymorphisms within the p53 gene were screened either by PCR (CA-repeat) or by direct sequencing as described above: a dinucleotide repeat polymorphism (Jones and Nakamura, 1992), a C/G polymorphism in intron 2 37 bp 3′ from exon 2 (Pignon *et al.*, 1994), a 16 bp insertion polymorphism in intron 3 15-23 bp 3′ of exon 3 (Lazar *et al.*, 1993), a C/G (Pro/Arg) polymorphism at codon 72 in exon 4 (Matlashewski *et al.*, 1987) and an A/G polymorphism in intron 6 61 bp 3′ of exon 6 (Chumakov and Jenkins, 1991).

**Figure 1** Examples of autoradiographs of the genotypic fingerprinting with highly polymorphic di- and trinucleotide repeats of the tumours with exactly the same mutation. (**a**) CTG B37, (**b**) DM, (**c**) D2S123. Lanes 1-3, tumours with mutation at amino acids 273 (113T, 114T, 115T); lanes 4-6, tumours with mutation at amino acids 183 (110T, 110T second sample, 202T); lanes 7-12, control tumours (193T, 194T, 195T, 197T, 199T, 200T).

*Immunohistochemical detection of p53 antigen overexpression*

Clusters of tumour cells in touch preparations were stained for expression of p53 as described previously (Kovach *et al.*, 1991; Sommer *et al.*, 1992). Briefly, the touch preparations were incubated with each of three p53-specific monoclonal antibodies (PAb 1801, 0.5 $\mu$g ml$^{-1}$, Cambridge Research Biochemicals; PAb 240 1 $\mu$g ml$^{-1}$, Santa Cruz Biochemicals; and PAb 421, 1 $\mu$g ml$^{-1}$, Oncogene Sciences). Bound antibody was detected with an avidin–biotin complex Elite kit (Vector) as directed by the manufacturer. Normal mouse IgG reagent was used as a negative control; tumour samples with p53 missense mutations, which show strong nuclear staining with all three antibodies, were used as positive controls. Tumours with 5% or more cells expressing p53 to any of the antibodies were considered immunohistochemically positive. Each experiment was repeated at least once to verify the results.

*Statistical analysis*

The patterns of mutation in breast cancer for the different populations were compared by the Fisher exact test using the Stat X Act software package (Cytel).

### Results

*Frequency and allelic status of p53 gene mutations*

Sixteen mutations were detected among 27 breast cancers (59.3%) upon analysis of exons 4–10 and the adjacent intronic sequences of the p53 gene (Table I). There were eight missense mutations, two nonsense mutations, four frameshift microdeletions/microinsertions and two mutations that alter a consensus splice sequence (one single base change and one inframe microdeletion/insertion). In contrast to findings in the US Caucasian, US African–American and Austrian populations previously studied (Saitoh *et al.*, 1994; Blaszyk *et al.*, 1994; Hartmann *et al.*, 1995*a*), in which wild-type sequence was absent from virtually all mutated tumours, 5 of 16 mutations were heterozygous in all cell clusters examined. Two other tumours showed a heterozygous pattern in at least one of the investigated tumour cell clusters.

*Clustering of p53 mutations*

Recurrent mutations were found at two sites. A transition at the dinucleotide CpG produced an arginine to histidine substitution at codon 273 in three tumours (113T, 114T and 115T) and a C:G→G:C transversion produced a nonsense mutation at codon 183 in two tumours (110T and 202T, Table I). To exclude the possibility of sample duplication or other artefact, six highly polymorphic di- and trinucleotide repeats in various regions of the genome were investigated in these five tumours by PCR. The three tumours with a mutation at amino acid 273 (113T, 114T and 115T) differed in at least three of the six polymorphic alleles, demonstrating that the mutations are independent. Investigation of the two tumours with the same mutation at amino acid 183 showed different alleles in three of six repeats (Figure 1). Thus, five independent mutations occurred at two mutational hotspots, making up 31% of 16 mutations found. Whereas, a mutation at amino acid 273 is a common change in breast and other types of cancer, only one mutation at amino acid 183 among 1965 p53 mutations has been reported among various cancers (De Vries *et al.*, 1996). There was no constitutional DNA available, so the possibility of germline mutations cannot be formally excluded. However, to exclude the possibility that the individuals with these mutations in our study had common ancestors, several polymorphisms were investigated *within* the p53 gene (see Materials and methods and Table III). Two polymorphisms were informative. A highly polymorphic dinucleotide repeat within p53 gene and a common polymorphism at amino acid 72 indicated that all five tumours with independent mutations were associated with different haplotypes. Furthermore, there was no family history of cancer in these patients.

*Pattern of mutation*

There were 16 independent mutations among 27 tumours. Because most studies of p53 gene mutations are limited to exons 5–9, for comparisons of patterns of mutations among different populations, we analysed the 15 mutations within this region of the gene. Mutations were classified into eight groups: deletions/insertions, G:C→A:T transitions at the dinucleotide CpG, G:C→A:T transitions not at CpG,

**Table I** Mutations in exons 2–11 in the p53 gene in primary breast cancers from Hirosaki, Japan

| Tumour no. | Age | Stage[a] | Exon/intron | Nucleotide change[b] | Structural change codon/amino acid | Immunohistochemistry: nuclear reactivity with antibody[c] PAb1801 | PAb240 | PAb421 | Mutation type | Allelic status[d] |
|---|---|---|---|---|---|---|---|---|---|---|
| 194T | 61 | IIIA | E6 | TGG(C→T)CCC | 189 A→V | – | + | – | Missense | Heterozygous |
| 204T | NA | NA | E6 | AGT(G→A)TGG | 216 V→M | + | + | + | Missense | Heterozygous |
| 199T | 57 | IIIA | E6 | CCT(A→G)TGA | 220 Y→C | + | + | + | Missense | Hemi/ Heterozygous[f] |
| 70T | 47 | I | E7 | GTA(A→C)CAG | 239 N→T | + | + | + | Missense | Hemizygous |
| 113T | 46 | IIA | E8 | TGC(G→A)TGT | 273 R→H | + | – | – | Missense | Hemizygous |
| 114T | 81 | IIIA | E8 | TGC(G→A)TGT | 273 R→H | + | + | + | Missense | Hemizygous |
| 115T | 41 | IIB | E8 | TGC(G→A)TGT | 273 R→H | – | – | – | Missense | Hemizygous |
| 69T | 67 | IIIA | E8 | ACA(G→A)AGG | 285 E→K | + | + | – | Missense | Hemizygous |
| 453T[e] | NA | NA | E4 | ACT (TGCACGg→a)tca | 124–125 7 bp deletion 1 bp insertion | – | + | – | Deletion (IF) splice site | Hemizygous |
| 193T | 60 | IIB | E5 | TCA(→CA)ACA | 131 2 bp insertion | – | + | – | Insertion (FS) | Heterozygous |
| 110T | 83 | IIA | E5 | GCT(C→G)AGA | 183 S→stop | – | + | – | Nonsense | Hemizygous |
| 202T | NA | NA | E5 | GCT(C→G)AGA | 183 S→stop | – | + | – | Nonsense | Hemizygous |
| 195T | 40 | IIA | E7 | ACT(→T)ACA | 236 1 bp insertion | – | + | – | Insertion (FS) | Hemizygous |
| 197T | 63 | IIIB | E7 | TGT(GTA→TT) ACA | 238 3 bp deletion 2 bp insertion 236 | – | + | – | Deletion/ insertion (FS) | Heterozygous |
| 71T[e] | 66 | IIB | E9 | ACC(AGCTC)CTC | 313–14 5 bp deletion | + | + | – | Deletion (FS) | Hemi/ heterozygous[f] |
| 200T[e] | 62 | IIA | I9 | CAG(g→t)tac | First 5′ base of intron 9 | + | + | – | Splice site | Heterozygous |

[a]American Joint Committee on Cancer (3rd edn). [b]Small characters are intronic sequence. [c]Tumours with 5% or more cells expressing p53 to any of the three antibodies were considered immunohistochemically positive. [d]The hemi- or heterozygous status of a tumour cell cluster was judged on the basis of the presence or absence of wild-type sequence at the site of the mutation. [e]Tumour cell clusters were either hemizygous or heterozygous for the p53 mutation. [f]Previously reported in Hartmann *et al.* (1995b). IF, inframe; FS, frameshift.

A:T→G:C transitions, and the four types of transversions. The pattern of mutation in the Hirosaki population was compared with our Midwestern white population (Sommer et al., 1992; Saitoh et al., 1994), a US African–American population from Detroit (Blaszyk et al., 1994), to pooled data from several western European populations (Coles et al., 1992; Mazars et al., 1992; Andersen et al., 1993; Thorlacius et al., 1993) and to two populations from Tokyo and Tokushima (Tsuda et al., 1993; Sasa et al., 1993). Pairwise comparisons among populations did not show statistically significant differences in patterns of p53 mutations between the Hirosaki sample and others (Table II).

*Immunohistochemistry*

Touch preparations were stained with three monoclonal p53 antibodies, PAb 1801, PAb 240 and PAb 421 (Table I). Among eight tumours with a missense mutation present, six were positive for p53 expression as assessed by nuclear staining with PAb 1801. Five of these also stained with PAb 240 and four with PAb 421. Only one of the three tumours with a missense mutation at codon 273 (114T) stained with all three antibodies; one tumour stained with PAb 1801 only (113T) and tumour 115T did not stain with any of the three antibodies. Eight tumours contained a null mutation (nonsense, frameshift or splice site mutations). Six of these did not react with PAb1801 and PAb 421 as expected for tumours with null mutations. The other two, 71T and 200T, which contained a frameshift deletion towards the C-terminal end of the gene and a splice site mutation in intron 9 respectively, reacted with PAb 1801 but not PAb 421, suggesting overexpression of a portion of the p53 protein.

### Discussion

*Frequency of p53 mutation*

Although the number of tumours investigated in this study is low, the high frequency of mutations in the p53 gene (55.5% in exons 5–9) is striking when compared with the frequencies of p53 mutations in the same exons in breast cancers in various European populations (pooled data; 21.6%; $P=0.0002$), in a US Midwestern white population (29.9%; $P=0.02$), in a US African–American population from Detroit (34%; $P=0.08$) and in Japanese women from Tokyo (24.6%; $P=0.002$) and from Tokushima (24.6%; $P=0.007$) (references in Table II). Although an unknown bias in sample collection among the various populations cannot be ruled out, this is unlikely. No statistically significant differences in various tumour parameters, including tumour size, stage, lymph node involvement and histological type were observed between the Hirosaki and the US patients, and the same techniques were used to detect and confirm mutations.

Oncoproteins from several DNA tumour viruses, such as the SV40 large T antigen, the adenovirus E1B and papillomavirus E6 protein are known to functionally inactivate wild-type p53 protein by binding to or by degrading the protein. The cellular oncogene *MDM2*, which is frequently amplified in sarcomas, is believed to decrease p53 function by binding to wild-type p53 (Momand et al., 1992; Oliner et al., 1993). If p53 mutagenesis is but one of several mechanisms responsible for breast carcinogenesis, the high frequency of p53 mutations in a low-risk group such as women from Hirosaki raises the possibility that in this group p53 mutagenesis is a predominate factor in breast cancer development whereas in other cohorts different mechanisms are likely to account for the higher proportion of breast cancer.

We are studying additional patients to confirm the difference in frequencies of p53 gene mutations between southern and northern Japanese populations and to determine if the mutations at codons 183 and 273 are favoured targets in the northern group.

*Allelic status of the mutations*

Another intriguing characteristic of p53 mutations in breast cancers of Japanese women from Hirosaki revealed by our

**Table II** Mutations in exons 5–9 of the p53 gene in breast cancers from Japanese, US and European patients

| Origin of patients | No. of mutations (% of tumours) | Deletion/insertion | Transitions G:C→A:T CpG | G:C→A:T non-CpG | A:T→G:C | Transversions G:C→C:G | G:C→T:A | A:T→C:G | A:T→T:A |
|---|---|---|---|---|---|---|---|---|---|
| Japan, Hirosaki | 15 (55.6) | 4 | 3 | 3 | 1 | 2 | 1 | 1 | 0 |
| Midwest US Rural White[a] | 29 (29.9) | 11 | 6 | 6 | 1 | 2 | 0 | 1 | 2 |
| US African American[b] | 16 (34.0) | 1 | 5 | 2 | 5 | 1 | 1 | 1 | 0 |
| Europeans[c] | 109 (21.6) | 14 | 26 | 19 | 9 | 7 | 19 | 11 | 4 |
| Japan, Tokyo[d] | 29 (24.6) | 6 | 5 | 6 | 7 | 0 | 1 | 2 | 2 |
| Japan, Tokushima[e] | 16 (24.6) | 1 | 5 | 2 | 2 | 1 | 1 | 1 | 3 |

[a]Sommer et al. (1992); Saitoh et al. (1994). [b]Blaszyk et al. (1994). [c]Pooled data from Scotland: Coles et al. (1992); France: Mazars et al. (1992); Norway: exons 5–8, Andersen et al. (1993); Iceland: exons 5,7,8, Thorlacius et al. (1993). All pairwise comparisons of the pattern in these cohorts were not statistically significant. [d]Tsuda et al. (1993). [e]Sasa et al. (1993) (exons 5–8).

**Table III** Comparison of the samples with the mutations at amino acids 183 and 273

| Tumour | Structural change codon/ amino acid | Data from Hirosaki clinical | Received | Date of surgery | Age | Immunohistochemistry PAb1801 | PAb240 | PAb241 | p53 gene polymorphism at amino acid 72 |
|---|---|---|---|---|---|---|---|---|---|
| 113 | 273 R→H | Solid tubular T2N0M0 (II) E, P→not done | 9/6/91 | 8/10/88 | 46 | + | + | – | Pro |
| 114 | 273 R→H | Solid tubular T1N0M0 (I) E–, P1 | 9/6/91 | 4/16/91 | 81 | + | + | + | Arg |
| 115 | 273 R→H | Scirrhous (T3N2M0 (III) E+, P+ | 9/6/91 | 5/31/91 | 41 | – | – | – | Pro/Arg |
| 202 | 183 S→stop | Solid tubular T2N1M0 E, P→ not done | 2/17/92 | NA | NA | – | + | – | Arg |
| 110 | 183 S→stop | Solid tubular T2N0M0 (II) E+, P+ | 9/6/91 | 7/3/91 | 83 | – | – | – | Arg |

Highly polymorphic repeats: 113T, 114T, 115T are different from each other in three repeats: 202T is different in three repeats from 110T. Polymorphisms within the p53 gene: insertion polymorphism in intron 3 and the polymorphisms in intron 2 and intron 6 are not informative; polymorphism at amino acid 72 is not informative for 110T and 202T; showed independence of mutation for 113T, 114T, 115T and showed that 113T and 114T are presumably not in the germ line; CA repeat within p53 showed that the mutations in 113T, 114T, 115T, 110T and 202T are independent and not in the germ line.

analyses is a high frequency of heterozygous rather than hemizygous mutations in this cohort (Table I). Seven of 16 mutations were heterozygous, i.e. had equal representation of wild-type and mutant sequence on genomic analysis. This high frequency of heterozygosity was statistically significant ($P < 0.05$) when compared with all non-Japanese populations analysed by us (Saitoh *et al.*, 1994; Blaszyk *et al.*, 1994; Hartmann *et al.*, 1995*a*). This observation raises the possibility that Japanese women have greater genomic stability, at least with respect to allelic deletion of the p53 gene, than various Western populations.

*Clustering of p53 mutations*

In the present sample, independent recurrent mutations were found at two sites (Table III). Three independent missense mutations occurred at amino acid 273 (G:C→A:T). This is a known mutational hotspot occurring at a CpG site coding for an amino acid that is conserved in all known p53 sequences (Soussi *et al.*, 1990). This degree of conservation extends over 1.6 billion years of evolutionary divergence. In a database of 1965 p53 mutations (De Vries *et al.*, 1996), this mutation occurs 112 times (7.3%). Among 186 breast cancer mutations, there are seven mutations at codon 273 (3.8%). There are several tumour types in which this G:C→A:T mutation occurs very frequently, comprising 50% of all mutations in thyroid cancers, 22.7% in pancreatic cancer and 15.9% in brain tumours. In contrast to this mutational hotspot, mutations at codon 183 are extremely rare. Only one other nonsense mutation in a bladder cancer has been reported at this location (Sidransky *et al.*, 1991). This 'clustering' of mutations, rather than the mutational pattern observed could point to a common mutagenic effect within this Japanese cohort.

*Pattern of p53 mutation*

Differences in patterns of p53 gene mutations in several types of cancer appear to reflect the effects of specific mutagens. Examples are the relationships among G:C→T:A transversions, mutagens in cigarette smoke and lung cancer (Caron de Fromentel and Soussi, 1992; Chiba *et al.*, 1990); G:C→T:A transversions in codon 249 of the p53 gene, aflatoxin B1 exposure and hepatocellular carcinomas (Bressac *et al.*, 1991; Hsu *et al.*, 1991); and C:G→T:A transitions and CC:GG→TT:AA tandem dipyrimidine transitions and multiple mutations, UV-B radiation and squamous and basal cell carcinomas of the sun-exposed skin (Brash *et al.*, 1991; Moles *et al.*, 1993).

In these cancers, analysis of mutations in the p53 gene confirms associations previously recognised by classical epidemiological studies. Identification of causative mutagens in these tumours may have succeeded because one mutagen predominates in each instance. In breast cancer, the risk of disease varies at least 4-fold among different populations. Some low-risk populations such as the Japanese have a marked increase in risk of breast cancer when they immigrate to a high-risk area such as the US (Stemmermann, 1991). Classical epidemiological studies have not yet identified unequivocally a mutagen associated with a high risk of breast cancer in any population, perhaps because different mutagens predominate in different populations. If so, different high-risk populations might have different patterns of p53 mutations since the patterns that mutagens produce are highly variable. Several populations at high risk of breast cancer have been shown to have different patterns of p53 gene mutation. The pattern of mutation in Midwest Caucasian women differs significantly from that in Scottish, Austrian and African-American females. The main differences between the populations are an abundance of microdeletions in the US Caucasian group (Sommer *et al.*, 1992; Saitoh *et al.*, 1994), a high frequency of G:C→T:A transversions in the Scottish group (Coles *et al.*, 1992), a high frequency of A:T→G:C transitions in African-Americans (Blaszyk *et al.*, 1994), and an abundance of A:T→T:A transversions in patients from Graz, Austria (Hartmann *et al.*, 1995*a*).

The Hirosaki population described in this report has a pattern of mutation that does not have a predominant type of mutation compared with several high-risk groups. The pattern seen could reflect mutagenic exposure similar to other groups as modified by genetic factors affecting processes such as DNA repair and/or adduct formation. Alternatively, the pattern in Hirosaki patients could reflect the baseline (endogenous) pattern unmodified by exogenous mutagens. In high-risk populations, a dominant mutagen or mutagens may skew the endogenous pattern in ways that the resulting patterns among high-risk groups differ more from each other than from the endogenous pattern in low-risk (non-mutagen exposed) groups. It will be difficult but important to distinguish among these alternatives. Analysis of the pattern of p53 mutations in breast cancers in other low-risk populations is needed to determine if a single pattern is characteristic of these groups. We are studying Japanese breast cancer patients who are migrants to the US to investigate the influence of a different lifestyle on the pattern of p53 mutation.

*Immunohistochemistry*

There are numerous reports of correlations between immunohistochemically detectable p53 expression in the nucleus of malignant cells and the presence of a missense mutation in the p53 gene and between absence of p53 expression and absence of p53 mutation or presence of a null mutation (nonsense, frameshift and splice site mutation; reviewed in Harris and Hollstein, 1993). Our previous studies of US Caucasian (Saitoh *et al.*, 1994), US African-American (Blaszyk *et al.*, 1994) and Austrian (Hartmann *et al.*, 1995*a*) cohorts of breast cancers showed a significant relationship between nuclear expression of the p53 antigen and the presence of a 'missense-type' mutation (missense and inframe microdeletion/insertion). In the Hirosaki patients, although six of eight tumours with missense mutations reacted with PAb 1801, an anti-p53 antibody that consistently stains cells with missense p53 mutations (JM Cunningham, personal communication), three tumours with the same missense change at codon 273 had different staining patterns. One stained only with PAb 1801, one with both PAb 1801 and PAb 240 and one did not stain, supporting the clustering of three identical mutations at this particular codon, which is unlikely to be due to technical artefact. As we have found in studies of p53 mutations in other populations, the antibody PAb240 frequently reacts with cells hemizygous or heterozygous for p53 gene null mutations (JM Cunningham, personal communication). None of the 11 tumours in which we did not detect a p53 gene mutation stained with PAb1801 or PAb421, but two stained with PAb240. The basis of these aberrant findings remains unclear, but they raise the possibility of a biological difference among tumours in this population that modifies p53 expression compared with other cohorts previously studied by us with identical methodology.

**Abbreviations**

ddF, dideoxy fingerprinting; AR, androgen receptor gene; DM, myotonic dystrophy gene; aa, amino acid.

**Acknowledgements**

This work was supported by grant RO1-CA56881 (JSK), a grant from the Dr Mildred Scheel Foundation for Cancer Research (AH, HB) and grant CA15086 (Molecular Probe Core of the Mayo Comprehensive Cancer Center), from the National Cancer Institute, NIH, DHHS, Bethesda, MD, USA.

### References

ANDERSEN TI, HOLM R AND NESLAND JM. (1993). Prognostic significance of TP53 alterations in breast carcinoma. *Br. J. Cancer*, **68**, 440–448.

BLASZYK H, VAUGHN C, HARTMANN A, MCGOVERN RM, SCHROEDER JJ, CUNNINGHAM J, SCHAID D, SOMMER SS AND KOVACH JS. (1994). Novel pattern of p53 gene mutations in an American black cohort with high mortality from breast cancer. *Lancet*, **343**, 1195–1197.

BLASZYK H, HARTMANN A, SCHROEDER JJ, MCGOVERN RM, SOMMER SS AND KOVACH JS. (1995). Rapid and efficient screening for p53 gene mutations by dideoxyfingerprinting (ddF). *Biotechniques*, **18**, 256–260.

BORING CC, SQUIRES TS AND TONG T. (1993). Cancer statistics, 1993. *CA Cancer J. Clin.*, **43**, 7–26.

BRASH DE, RUDOLPH JA, SIMON JA, LIN A, MCKENNA GJ, BADEN HP, HALPERIN AJ AND PONTEN J. (1991). A role for sunlight in skin cancer: UV-induced p53 mutations in squamous cell carcinoma. *Proc. Natl Acad. Sci. USA*, **88**, 10124–10128.

BRESSAC B, KEW M, WANDS J AND OZTURK M. (1991). Selective G to T mutations of p53 gene in hepatocellular carcinoma from southern Africa. *Nature*, **350**, 429–431.

BROOK JD, MCCURRACH ME, HARLEY HG, BUCKLER AJ, CHURCH D, ABURATANI H, HUNTER K, STATON VP, THIRION J, HUDSON T, SOHN R, ZEMELMAN B, DAIVES J, SHELBOURNE P, BUXTON J, JONES C, JUVONEN V, JOHNSON K, HARPER PS, SHAW DJ AND HOUSEMAN DE. (1992). Molecular basis of myotonic dystrophy: expansion of a trinucleotide (CTG) repeat at the 3′ end of a transcript encoding a protein kinase family member. *Cell*, **68**, 799–808.

CARON DE FROMENTEL C AND SOUSSI T. (1992). TP53 tumour suppressor gene: A model for investigating human mutagenesis. *Genes Chrom. Cancer*, **4**, 1–15.

CHIBA I, TAKAHASHI T, NAU MM, D'AMICO D, CURIEL DT, MITSUDOMI T, BUCHHAGEN DL, CARBONE D, PIANTADOSI S, KOGA H, REISSMAN PT, SLAMON DJ, HOLMES EC AND MINNA JD. (1990). Mutations in the p53 gene are frequent in primary, resected non-small cell lung cancer. *Oncogene*, **5**, 1603–1610.

CHUMAKOV PM AND JENKINS JR. (1991). BstNI/NciI polymorphism of the human p53 gene. *Nucleic Acids Res.*, **19(24)**, 6969.

COLEMAN MP, ESTEVE J, DAMIECKI P, ARSLAN A AND RENARD H. (1993). *Trends in cancer incidence and mortality*, IARC Scientific Publications: Lyon.

COLES C, CONDIE A, CHETTY U, STEEL CM, EVANS JH AND PROSSER J. (1992). p53 mutations in breast cancer. *Cancer Res.*, **52**, 5291–5298.

DE VRIES EMG, RICKE DO, DE VRIES TN, HARTMANN A, BLASZYK H, SOUSSI T, KOVACH JS AND SOMMER SS. (1996). Database of mutations in the p53 and APC tumour suppressor genes. *Human Mutation* (in press).

DUTTON CM, PAYNTON C AND SOMMER SS. (1993). General method for amplifying regions of very high G + C content. *Nucleic Acids. Res.*, **21(12)**, 2953–2954.

HARRIS CC AND HOLLSTEIN M. (1993). Clinical implications of the p53 tumour-suppressor gene. *N. Engl. J. Med.*, **329**, 1318–1327.

HARTMANN A, ROSANELLI G, BLASZYK H, CUNNINGHAM J, MCGOVERN RM, SCHROEDER JJ, SCHAID D, KOVACH JS AND SOMMER SS. (1995*a*). Novel pattern of p53 mutation in breast cancers from Austrian women. *J. Clin. Invest.*, **95**, 686–689.

HARTMANN A, BLASZYK H, MCGOVERN RM, SCHROEDER JJ, CUNNINGHAM J, DE VRIES EMG, KOVACH JS AND SOMMER SS. (1995*b*). p53 gene mutations inside and outside of exons 5–8: the patterns differ in breast and other cancers. *Oncogene*, **10**, 681–688.

HSU IC, METCALF RA, SUN T, WELSH JA, WANG NJ AND HARRIS CC. (1991). Mutational hotspot in the p53 gene in human hepatocellular carcinoma. *Nature*, **350**, 427–428.

JONES MH AND NAKAMURA Y. (1992). Detection of loss of heterozygosity at the human TP53 locus using a dinnucleotide repeat polymorphism. *Genes Chrom. Cancer*, **5**, 89–90.

JONES PA, BUCKLEY JD, HENDERSON BE, ROSS PK AND PIKE MC. (1991). From gene to carcinogen: A rapidly evolving field in molecular epidemiology. *Cancer Res.*, **51**, 3617–3620.

KOVACH JS, MCGOVERN RM, CASSADY JD, SWANSON SK, WOLD LE, VOGELSTEIN B AND SOMMER SS. (1991). Direct sequencing from touch preparations of human carcinomas: Analysis of p53 mutations in breast carcinomas. *J. Natl Cancer Inst.*, **83**, 1004–1009.

KOVACH JS, HARTMANN A, BLASZYK H, CUNNINGHAM J, SCHAID D AND SOMMER SS. (1996). Detection of p53 gene mutations in breast cancer by highly sensitive methods can provide important prognostic information. *Proc. Natl. Acad. Sci. USA*, (in press).

LA SPADA AR, WILSON EM, LUBAHN DB, HARDING AE AND FISCHBECK KH. (1992). Androgen receptor gene mutations in X-linked spinal and bulbar muscular atrophy. *Nature*, **352**, 77–79.

LAZAR V, HAZARD F, BERTIN F, JANIN N, BELLET D AND BRESSAC B. (1993). Simple sequence repeat polymorphism within the p53 gene. *Oncogene*, **8**, 1703–1705.

LI SH, MCINNIS MG, MARGOLIS RL, ANTONARAKIS SE AND ROSS CA. (1993). Novel triplet repeat containing genes in human brain: cloning, expression, and length polymorphisms. *Genomics*, **16**, 572–579.

MATLASHEWSKI GJ, TUCK S, PIM D, LAMB P, SCHNEIDER J AND CRAWFORD LV. (1987). Primary structure polymorphism at amino acid residue 72 of human p 53. *Mol. Cell. Biol.*, **7**, 961–963.

MAZARS R, SPINARDI L, BENCHEIKH M, SIMONY-LAFONTAINE J, JEANTEUR P AND THEILLET C. (1992). p53 mutations occur in aggressive breast cancer. *Cancer Res.*, **52**, 3918–3923.

MOLES JP, MOYRET C, BUILLOT B, JEANTEUR P, GUILHOU JJ, THEILLET C AND BASSET-SEGUIN N. (1993). p53 gene mutations in human epithelial skin cancers. *Oncogene*, **8**, 583–588.

MOMAND J, ZAMBETTI GP, OLSON DC, GEORGE D AND LEVINE AJ. (1992). The *mdm-2*oncogene product forms a complex with the p53 protein and inhibits p53-mediated transactivation. *Cell*, **69**, 1237–1245.

OLINER JD, PIETENPOL JA, THIAGALINGAM S, GYURIS J, KINZLER KW AND VOGELSTEIN B. (1993). Oncoprotein MDM2 conceals the activation domain of tumour suppressor of p53. *Nature*, **362**, 857–860.

PIGNON JM, VINATIER I, FANEN P, JONVEAUX P, TOURNILHAE O, IMBERT M, ROCHANT H AND GOOSENS M. (1994). Exhaustive analysis of the p53 gene coding sequence by dematuring gradient gel electrophoresis: application to the detection of point mutations in acute leukemias. *Human Mutation*, **3**, 126–132.

SAITOH S, CUNNINGHAM J, DE VRIES EMG, MCGOVERN RM, SCHROEDER JJ, HARTMANN A, BLASZYK H, WOLD LE, SCHAID D, SOMMER SS AND KOVACH JS. (1994). p53 gene mutations in breast cancers in Midwestern US women: null as well as missense-type mutations are associated with poor prognosis. *Oncogene*, **9**, 2869–2875.

SARKAR G, YOON H-S AND SOMMER SS, (1992). Dideoxy fingerprinting (ddF): A rapid and efficient screen for the presence of mutations. *Genomics*, **13**, 441–443.

SASA M, KONDO K, KOMAKI K, UYAMA T, MORIMOTO T AND MONDEN Y. (1993). Frequency of spontaneous p53 mutations (CpG site) in breast cancer in Japan. *Breast Cancer Res. Treat.*, **27**, 247–252.

SHIELDS PG AND HARRIS CC. (1991). Molecular epidemiology and the genetics of environmental cancer. *JAMA*, **266**, 681–687.

SIDRANSKY D, VON ESCHENBACH A, TSAI YC, JONES P, SUMMERHAYES I, MARSHALL F, PAUL M, GREEN P, HAMILTON SR, FROST P AND VOGELSTEIN B. (1991). Identification of p53 gene mutations in bladder cancers and urine samples. *Science*, **252**, 706–709.

SOMMER SS. (1990). Mutagen test. *Nature*, **346**, 22–23.

SOMMER SS AND VIELHABER EL. (1994). Phage promoter-based methods for sequencing and screening for mutations. In *The Polymerase Chain Reaction*, Ferre F, RA Gibbs (eds). pp.214–220. Birkhauser: Boston.

SOMMER SS, CUNNINGHAM J, MCGOVERN RM, SAITOH S, SCHROEDER JJ, WOLD LE AND KOVACH JS. (1992). Pattern of p53 gene mutations in breast cancers of women of the midwestern United States. *J. Natl Cancer Inst.*, **84**, 246–252.

SOUSSI T, CARON DE FROMENTEL C AND MAY P. (1990). Structural aspects of the p53 protein in relation to gene evolution. *Oncogene*, **5**, 945–952.

STATXACT, ver 2.0, 1991. Cytel Software Corporation, Cambridge, MA.

STEMMERMANN GN. (1991). The pathology of breast cancer in Japanese women compared to other ethnic groups: a review. *Breast Cancer Res. Treat.*, **18**, 67–72.

STOFLET ES, KOEBERL DS, SARKAR G AND SOMMER SS. (1988). Genomic amplification with transcript sequencing. *Science*, **239**, 491–494

THORLACIUS S, BORRESEN A-L AND EYFJORD JE. (1993). Somatic p53 mutations in human breast carcinomas in an icelandic population: A prognostic factor. *Cancer Res.*, **53**, 1637–1641.

TSUDA H, IWAYA K, FUKUTOMI T AND HIROHASHI S. (1993). p53 mutations and c-erbB-2 amplificaton in intraductal and invasive breast carcinomas of high histologic grade. *Jpn. J. Cancer Res.*, **84**, 394–401.

WEISSENBACH J, GYAPAY C, DIB C, VIGNAL A, MORISSETTE J, MILLASSEAU P, VAYSSEIX G AND LATHROP M. (1992). A second generation linkage map of the human gene. *Nature*, **359**, 794–801.